# Cognitive compensatory mechanisms in normal aging: a study on verbal fluency and the contribution of other cognitive functions

**DOI:** 10.18632/aging.102040

**Published:** 2019-06-22

**Authors:** Lissett Gonzalez-Burgos, Juan Andrés Hernández-Cabrera, Eric Westman, José Barroso, Daniel Ferreira

**Affiliations:** 1 Department of Clinical Psychology, Psychobiology and Methodology, Faculty of Psychology, University of La Laguna, La Laguna, Tenerife, Spain; 2 Division of Clinical Geriatrics, Center for Alzheimer Research, Department of Neurobiology, Care Sciences, and Society, Karolinska Institutet, Stockholm, Sweden; 3 Department of Neuroimaging, Centre for Neuroimaging Sciences, Institute of Psychiatry, Psychology and Neuroscience, King's College London, London, UK

**Keywords:** verbal fluency, aging, differentiation, compensation, random forest

## Abstract

Verbal fluency has been widely studied in cognitive aging. However, compensatory mechanisms that maintain its optimal performance with increasing age are not completely understood. Using cross-sectional data, we investigated differentiation and dedifferentiation processes in verbal fluency across the lifespan by analyzing the association between verbal fluency and numerous cognitive measures within four age groups (N=446): early middle-age (32-45 years), late middle-age (46-58 years), early elderly (59-71 years), and late elderly (72-84 years). ANCOVA was used to investigate the interaction between age and fluency modality. Random forest models were conducted to study the contribution of cognition to semantic, phonemic, and action fluency. All modalities declined with increasing age, but semantic fluency was the most vulnerable to aging. The most prominent reduction in performance was observed during the transition from middle-age to early elderly, when cognitive variables stopped contributing (differentiation), and new cognitive variables started contributing (dedifferentiation). Lexical access, processing speed, and executive functions were among the most contributing functions. We conclude that the association between age and verbal fluency is masked by age-specific influences of other cognitive functions. Differentiation and dedifferentiation processes can coexist. This study provides important data for better understanding of cognitive aging and compensatory processes.

## Introduction

Cognitive decline is inherent to the normal aging process. Abilities such as executive functions, processing speed, memory, attention, and visuoconstructive and visuospatial functions decline with age [1–5]. Other functions such as crystallized abilities remain stable or even improve with age [1,6,7].

The effect of aging on language abilities has always attracted a great interest. Language is one of the most complex functions in humans, it is essential for the communication between people, and its impairment has traditionally been a subject of intense study [8]. Interestingly, perhaps due to its strong biological role, studies on normal aging have shown that some language abilities are quite resilient to the onslaught of aging. Comprehension, semantic abilities, and vocabulary remain rather stable or even improve with age [9,10]. Contrarily, other abilities such as verbal fluency and naming are among the most vulnerable cognitive functions to aging [11].

Cognitive tests of verbal fluency measure the ability to produce as many words as possible according to specific rules and a time limit. Phonemic fluency refers to the production of words beginning with a given letter (e.g. “F”). Semantic fluency refers to the production of words belonging to a semantic category (e.g. “animals”). Action fluency refers to the production of words belonging to the grammatical category of verbs (e.g. “to reflect”) [12]. Despite extensive research on the effect of age on verbal fluency, findings are not completely consistent. Numerous studies have shown that semantic fluency declines with age and phonemic fluency seems to be more stable [13–18]. However, contrary results are also common [19,20]. Research on action fluency in normal aging is scarce. Some studies showed lower word production with increasing age [20–22], while other studies showed similar levels of word production with increasing age [12,22,23]. The reason for these contradictory findings is partly related to methodological differences across studies such as the use of different study designs (longitudinal *vs.* cross-sectional), the sample (size, selection criteria, age groups, age span, etc.), and the statistical approach (correlation *vs.* means comparison *vs.* covariance analysis, etc.), among others. In addition, variation on the age span studied has implications beyond mere methodological differences because different compensatory mechanisms may be active and influence fluency performance differently at different ages.

However, compensatory mechanisms have not been investigated in detail. Understanding how diverse cognitive functions contribute to maintain an optimal performance in verbal fluency is of relevance. Previous studies have reported an association of semantic fluency with processing speed [17,24,25], lexical access [25–27], executive functions [26,28], and working memory [25]. Phonemic fluency has been reported to be associated with processing speed [17,24,25], attention [13,29], lexical access [27], executive functions [26,28–31], and memory [29,32]. Studies on action fluency did not find an association with episodic memory or picture naming [12,23]. Whether these associations contribute to compensatory effects across age is unknown. In addition, these associations may change with age. According to the “age differentiation hypothesis” [33], the organizational structure of cognitive abilities changes with age [34]. In particular, cognitive abilities shift from a differentiated condition at younger ages (abilities are separate systems: differentiation), into a dedifferentiated condition at older ages (abilities are more interrelated with each other: dedifferentiation) [35]. This higher intercorrelation proposed by the “age dedifferentiation hypothesis” is associated with reduced neural specificity to cognitive processes as a consequence of biological brain aging and increased interhemispheric activations [35–37]. However, studies addressing the dedifferentiation hypothesis of cognitive aging have generated inconsistent findings, probably due to differences in cognitive abilities assessed, age ranges of the included samples, and analytical techniques used across studies [38]. Further, very few studies have investigated differentiation and dedifferentiation processes on verbal fluency across the whole lifespan [1,17,29,31]. Advancing in our understanding of compensatory mechanisms, differentiation, and dedifferentiation processes is expected to have important implications. In clinical practice, this knowledge could contribute to reach a more accurate diagnosis of cognitive disorders, and could facilitate early and personalized therapeutic interventions. Scientifically, this knowledge may help to better understand age-related processes of the human brain, and its dynamic responses to both negative and positive influences.

The overall purpose of this cross-sectional study was to investigate how differentiation and dedifferentiation processes in verbal fluency are organized across the lifespan. Therefore, we investigated the association between performance in three components of verbal fluency (semantic, phonemic, and action) and performance in numerous non-fluency cognitive measures within different age groups from the early middle-age to the late elderly.

## RESULTS

In order to study the association between age and verbal fluency, the sample was divided into four equidistant age groups based on the own sample age distribution: early middle-age (32 to 45.9 years), late middle-age (46 to 58.9 years), early elderly (59 to 71.9 years), and late elderly (72 to 84.9 years). Table 1 shows the demographic characteristics of these age groups.

**Table 1:** Demographic characteristics and verbal fluency performance.

	**Early middle-age** **(n=79)**	**Late middle-age** **(n=143)**	**Early elderly** **(n=162)**	**Late elderly** **(n=62)**	
	**M(SD)/count(%)**	**M(SD)/count**	**M(SD)/count**	**M(SD)/count**	**p-value**
Age, years (min-max)	41.4 (2.8) ^a,b,c^ (32-45.9)	51.0 (3.9) ^b,c^ (46-58.9)	65.6 (3.4) ^c^ (59-71.9)	74.9 (2.3) (72-84.9)	<0.001
Sex (female, count (%))	43 (54.4)	79 (55.2)	91 (56.2)	32 (51.6)	0.943
Education level					
Illiteracy Unfinished primary studies Completed primary studies Completed secondary studies University studies	0 0 34 25 20	0 3 52 39 49	6 32 52 24 48	1 18 24 13 5	<0.001
WAIS-III Information	15.1 (5.9) ^a,c^	17.3 (5.8) ^b,c^	14.5 (6.3) ^c^	12.4 (5.9)	<0.001
Semantic fluency	22.6 (5.6) ^a,b,c^	22.2 (5.5) ^b,c^	18.2 (5.2) ^c^	15.7 (3.8)	<0.001
Phonemic fluency	34.8 (10.1) ^b,c^	37.2 (11.8) ^b,c^	28.1 (13.8)	25.8 (10.4)	<0.001
Action fluency	18.1 (6.8) ^b,c^	19.3 (7.6) ^b,c^	12.9 (7.4)	10.1 (4.9)	<0.001

WAIS-III: Wechsler Adult Intelligence Scale, Third edition.

^a^ Significantly different from Late middle-age.

^b^ Significantly different from Early elderly. ^c^ Significantly different from Late elderly.

### Age-related differences on verbal fluency

**Figure 1:**
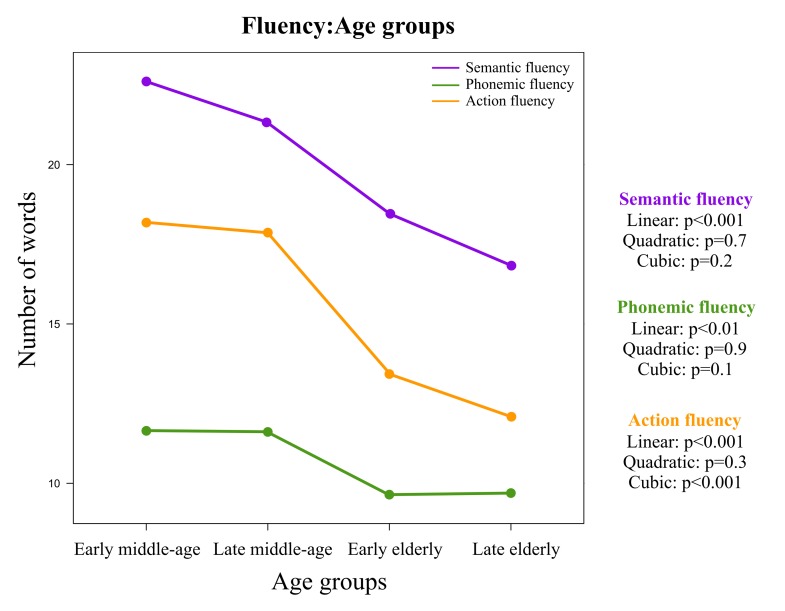
**The mixed ANCOVA model for age-related differences on verbal fluency**. The x-axis represents the age groups. The y-axis represents the number of words produced. The total number of words produced on phonemic fluency (F+A+S) was divided by three in order to allow comparability among the three fluency modalities (1 minute). P-values are reported for the estimation of linear, quadratic, and cubic effects from the trend analysis tested through the ANCOVA model. The lines represent the outcome from the mixed ANCOVA for age (between-subjects factor) and fluency modality (within-subjects factor) using cross-sectional data.

A mixed ANCOVA model was conducted to examine the interaction between age and verbal fluency. The age groups (4 levels: early middle-age, late middle-age, early elderly, and late elderly) served as the between-subject factor, and the verbal fluency task (3 levels: animals, phonemic, and action fluency) served as the within-subject factor. WAIS-III Information was included as a covariate. This model showed a significant interaction (F_(6, 882)_=7.8; p<0.001) (Figure 1). There is a clear association between age and verbal fluency, but this association is modulated by the fluency modality. The two middle-age groups do not differ with each other in performance on phonemic and action fluency (p>0.05), and perform rather similar on semantic fluency (p=0.048). However, the two middle-age groups always outperform the two elderly groups (p<0.01). In addition, the early-elderly group outperformed the late-elderly group, but only on semantic fluency (p<0.05) (Table 1). Due to this finding, the two middle-age groups were combined together and only three age groups were used for subsequent analyses (i.e. middle-age, early elderly, and late elderly) (Table 2).

**Table 2:** Demographic characteristics and verbal fluency performance in the three age groups.

	**Middle-age** **(n=222)**	**Early elderly** **(n=162)**	**Late elderly** **(n=62)**	
	**M(SD)**	**M(SD)**	**M(SD)**	**p-value**
Age, years (min-max)	47.6 (5.8) ^a,b^ (32-58)	65.6 (3.4) ^b^ (59-71)	74.9 (2.3) (72-84)	<0.001
Sex (female, n (%))	122 (55,0)	91 (56.2)	32 (51.6)	0.829
WAIS-III Information	16.5 (5.9) ^a,b^	14.5 (6.3) ^b^	12.4 (5.9)	<0.001
Semantic fluency	22.3 (5.5) ^a,b^	18.2 (5.2)	15.7 (3.8)	<0.001
Phonemic fluency	36.4 (11.2) ^a,b^	28.1 (13.8)	25.8 (10.4)	<0.001
Action fluency	18.9 (7.4) ^a,b^	12.9 (7.3)	10.1 (4.9)	<0.001

WAIS-III: Wechsler Adult Intelligence Scale, Third edition. Middle-age = Early middle-age + Late middle-age.

^a^ Significantly different from Early elderly.

^b^ Significantly different from Late elderly.

### Contribution of cognitive variables to verbal fluency by age groups

To assess whether the contribution of numerous cognitive variables to verbal fluency differs across age, a random forest regression model was performed separately for each of the three age groups (i.e. middle-age, early elderly, and late elderly). For a description of the cognitive variables (predictors) included in the random forests and their abbreviation please see “Neuropsychological assessment” in the Methods section as well as the Supplementary Table S1.

Table 3 shows that while similar cognitive abilities contributed to verbal fluency across age, some interesting differences can be observed.

**Table 3:** Contribution of cognitive variables to verbal fluency by age groups (random forest regression models).

	**Semantic fluency**									**Phonemic fluency**										**Action fluency**						
	**ME**	**EE**		**LE**		Pattern				**ME**		**EE**		**LE**		Pattern				**ME**	**EE**		**LE**		Pattern		
**Sample size, n**	222	162		62						222		162		62						222	162		62				
**Explained variance**	27%	33%		20%						35%		56%		39%						43%	47%		23%				
**Predictors**																											
BNT	22	20		8		S/Dif.				35		48		17		S.				26	24		8		S/Dif.		
PCV - Decision time	7			4		S.				5						Dif.									S.		
PCV - Motor time	19					Dif.				3		8		2		S.									S.		
PASAT	2	6		2		S.				5				1		S.									S.		
STROOP Words	9	16		7		S.				30		28		19		S.				29	15		24		S.		
STROOP Colors	11	14		18		S.				19		33		19		S.				21	9		10		S.		
STROOP Inhibition	3	15		4		S.				2		20		3		S.				9	6		3		S.		
TMT A	22	21		16		S.				19		22		14		S.				25	20		8		S/Dif.		
CTT - Part 1	5	25		19		S/Ded.				5		32		24		S/Ded.				17	37		24		S.		
CTT - Part 2	25	9		17		S.						31		13		Ded.				10	35		14		S.		
FRT	2	3				Dif.								3		Ded.				4					Dif.		
JLOT - First half	5	6				Dif.				2		8		5		S.					9		6		Ded.		
JLOT - Second half	5	6				Dif.				2				16		S/Ded.				8	8				Dif.		
Digit Span forward	5	12				Dif.				8		20		6		S.				24	10		2		S/Dif.		
Digit Span backward	4	13				Dif.				35		14		3		S/Dif.				27	2				Dif.		
Spatial Span forward	1					S/Dif.								6		Ded.				8	5				Dif.		
Spatial Span backward		6				S.				5				9		S.				2	10		12		S.		
LM A - Immediate	2	20		3		S.				5		9		4		S.				8	9		10		S.		
LM B1 - Immediate	13	13				Dif.				12		19				Dif.				25	11		17		S.		
LM B2 - Immediate	8	10				Dif.				7		14		15		S.				19	19		15		S.		
LM A - Delay	3	14		4		S.				5		9				Dif.				14	10		3		S/Dif.		
LM B - Delay	11	10				Dif.				7		16		3		S.				18	18		19		S.		
LM A - Recognition	3					Dif.				3		2		6		S.				3	3				Dif.		
LM B - Recognition	7	9				Dif.				16		13				Dif.				5	10		7		S.		
TAVEC 1st trial	5			7		S.						4				S.							1		S/Ded.		
TAVEC Learning	15	12		22		S.						7				S.					6				S.		
TAVEC Short delay	6	11		4		S.						8				S.					1		3		Ded.		
TAVEC Short delay-Clues		10		3		Ded.										S.									S.		
TAVEC Long delay		1		6		Ded.						4				S.					5				S.		
TAVEC Long delay-Clues	4	8		2		S.						3				S.				2	9				Dif.		
TAVEC Intrusions	2	8				Dif.				1		4				Dif.				1					S/Dif.		
TAVEC Intrusions-Clues	2					Dif.				3		7				Dif.				1			14		S/Ded.		
TAVEC Perseverations	1			3		S.				5						Dif.				8					Dif.		
TAVEC Recog. Correct		1				S.										S.									S.		
TAVEC Recog. False Positive				5		Ded.						5		2		Ded.				4					Dif.		
VR I – Total score	3	9				Dif.				3		9		6		S.				4	17		7		S.		
VR II – Total score	5			7		S.						9		10		Ded.				3	14		12		S.		
VR-Copying						S.				4		3				Dif.				6	4		5		S.		
VR Total Recog.	7	9		8		S.						10		10		Ded.					16		8		Ded.		
VR False Positive	6			7		S.				1				5		S.					1		8		Ded.		
VR Visual discrimination						S.				4		2				Dif.				7			4		S.		
Luria’s HAM Right	3	7		5		S.				11		10				Dif.				7	16		1		S.		
Luria’s HAM Left	8			7		S.				3						Dif.				6	9				Dif.		
Luria’s – Motor coordination				12		Ded.				5		3		18		S/Ded.				3	7		14		S.		
Block Design	6			1		S.				21		9		8		S/Dif.				34	5				Dif.		
Empty cells	8	15		19						13		11		18						12	12		18				
Associations	37	30		26						32		34		27						33	33		27				
Stable	53%									51%										53%							
Differentiation	36%									29%										33%							
Dedifferentiation	11%									20%										14%							
*Importance*	NC				<10			10			-		19		20		-			29				>30			

ME = Middle-age, EE = Early elderly, LE = Late elderly. The explained variance is the total cumulative variance explained by all the predictors in the model. BNT = Boston Naming Test (spontaneous responses). PCV = PC-Vienna System. PASAT = Paced Auditory Serial Addition Test. TMT A = Trial Making Test A. CTT = Color Trails Test. FRT = Facial Recognition Test. JLOT = Judgment of Line Orientation Test. LM = Logical Memory. VR = Visual Reproduction Test. Luria’s HAM = Luria’s Premotor Functions, Hand Alternative Movements. The numbers inside the cells in the “Predictors” area show the importance of each variable in predicting the outcome variable, where the higher the value the higher the importance. The importance is calculated as the relative error in the prediction when a given predictor is excluded from the model. Gray-shaded cells denote that these variables were not important in the model. S.= Stable; Dif. = Differentiation; Ded. = Dedifferentiation; S/Dif. = Stable/Differentiation; S/Ded. = Stable/Dedifferentiation. NC = no contribution. Empty cells = the total number of variables without any contribution. Associations = the total number of variables that are important to predicting verbal fluency.

Regarding semantic fluency, the most important variables in predicting performance in the middle-age group were CCT, TMT, BNT, PC-Vienna, Logical Memory (Immediate and Delayed), and TAVEC (Learning). In the early elderly group, the most important variables in predicting performance were CCT, TMT, BNT, Logical Memory (Immediate and Delayed), and Stroop. In the late elderly group, the most important variables in predicting performance were CTT, TMT, TAVEC (Learning), Stroop, and Luria’s motor coordination.

Regarding phonemic fluency, the most important variables in predicting performance in the middle-age group were BNT, Stroop, TMT, Digit Span backward, Logical Memory (Immediate), and Block Design. In the early elderly group, the most important variables in predicting performance were BNT, Stroop, TMT, Digit Span forward, and CTT. In the late elderly group, the most important variables in predicting performance were BNT, Stroop, Logical Memory (Immediate), CTT, Luria’s motor coordination, and JLOT.

Regarding action fluency, the most important variables in predicting performance in the middle-age group were TMT, BNT, Logical Memory, Stroop, Block Design, and Digit Span. In the early elderly group, the most important variables in predicting performance were TMT, BNT, Logical Memory, CTT, Visual Reproduction, and Luria’s hand alternative movements. In the late elderly group, the most important variables in predicting performance were Logical Memory, Stroop, CTT, Visual Reproduction, Luria’s motor coordination, TAVEC (Intrusions), and Spatial Span.

Virtually the same results were obtained when including WAIS-III Information and sex as extra predictors in order to investigate their potential confounding effect (data not shown).

### Differentiation, dedifferentiation, and stability patterns across age

Three different patterns can be observed in regard to the contribution of cognitive abilities to verbal fluency with increasing age. A differentiation pattern can be observed when cognitive variables stop contributing with increasing age. The dedifferentiation pattern is observed when variables start contributing with increasing age. A stability pattern is seen when the contribution of the variables remains stable across age. In some cases, a combination of these patterns can also be observed in the same variable. We classified as stable/differentiation and stable/dedifferentiation those variables that, despite showing mostly a stability pattern, stop or start contributing with increasing age, respectively. More detail on the procedure to ascertain these patterns is provided in Supplementary Table S2.

Overall, semantic fluency was associated with less cognitive variables with increasing age, indicating a differentiation pattern with aging (Table 3). This is explained because although stability in the associations was observed in 53% of the variables, the percentage of variables showing a differentiation pattern (36%) exceeded the percentage of variables showing a dedifferentiation pattern (11%). In particular, several recall variables of Logical Memory stop contributing to semantic fluency after the early elderly, together with JLOT, PC-Vienna, Digits, Visual Reproduction, and FRT (differentiation). On the other hand, several delayed recall variables of TAVEC as well as Luria’s motor coordination start contributing to semantic fluency in the late elderly (dedifferentiation). Variables with stable contribution are shown in Table 3.

Phonemic fluency showed more stability in the number of cognitive associations with increasing age. The reason for this is that stability in the associations was observed in 51% of the variables, and the percentage of variables showing a differentiation pattern (29%) was rather comparable to the percentage of variables showing a dedifferentiation pattern (20%) with aging, thus cancelling each other. We observed that several recall variables of Logical Memory stop contributing to phonemic fluency after the early elderly, together with TAVEC errors, Visual Reproduction copy and visual discrimination, and Luria’s hand alternative movements (differentiation). In contrast, several delayed recall variables of Visual Reproduction, TAVEC, FRT and CTT start contributing to phonemic fluency in the late elderly (dedifferentiation). Variables with stable contribution are shown in Table 3.

The results in action fluency are a combination of the patterns described above for semantic fluency and phonemic fluency. Stability in the number of cognitive associations prevailed from middle-age to early elderly, while a reduction in the contributing cognitive variables was observed when reaching the late elderly group. This is explained because stability in the associations was observed in a slightly superior proportion of variables as compared with the other two fluency modalities (53%), but the proportion of variables showing a differentiation pattern (33%) exceeded the number of variables showing a dedifferentiation pattern (14%). In particular, several variables of TAVEC stop contributing to action fluency after the early elderly, together with Luria’s motor coordination, JLOT, Block design, Spatial span forward, Digit span backward and FRT (differentiation). The Delayed recall variables of Visual Reproduction and TAVEC start contributing to action fluency in the late elderly, together with JLOT (dedifferentiation).

## DISCUSSION

The overall purpose of this study was to investigate how differentiation and dedifferentiation processes in verbal fluency are organized across the lifespan (32 to 84 years). Using cross-sectional data, we investigated the association between performance in three components of verbal fluency (semantic, phonemic, and action) and performance in numerous non-fluency cognitive measures within different age groups from the early middle-age to the late elderly.

Although we found a lower word production with increasing age in the three fluency modalities, age showed a stronger association with semantic fluency than with the other two modalities. The most prominent reduction in performance was observed between the middle-age and the early elderly in the three modalities. At that point in time, a high number of cognitive variables stopped contributing specially to semantic and action fluency. Despite potentially compensatory dedifferentiation patterns in the three modalities, a stronger differentiation process was observed in the three modalities.

### The association between age and verbal fluency

Semantic fluency showed a lineal and progressive reduction throughout the whole age range investigated in this study. Other studies including cohorts with a wide range of age have also observed a linear association between age and semantic fluency [39–42]. Some authors have also reported relative stability until the age of 60 [2,16,17,43,44], followed by a decline in performance [1,16,20,44–49]. Therefore, the association between age and semantic fluency is a quite well established finding, although negative reports also exist [30]. Phonemic fluency was rather stable during the middle-age, followed by a drop during the early elderly that seems to get stabilised in the late elderly. Despite these dynamics, our models showed that the linear trend was the best fit (as compared with quadratic and cubic trends). A linear association between age and phonemic fluency has also been observed in previous studies [41,42,50]. Similar to our results, several studies have shown certain stability until the ages of 60-65 years [2,20,43,44], followed by decline [20,39,41,47,49]. However, no association between age and phonemic fluency has also been reported [13,16,26,31,48]. Regarding action fluency, a cubic trend was the best fit in our data. We observed a plateau of high performance during the middle-age, with a drop during the early elderly, and a trend for stability in performance during the late elderly. Previous studies only included elderly individuals and did not found an association between age and action fluency [12,23]. Such finding is in line with the trend for stability in our older age strata.

Fewer studies have simultaneously compared the association between age and the different fluency modalities in the same cohort and statistical model. Indeed, these studies have only compared semantic and phonemic fluency, whereas no data existed on action fluency to the present date. Although we found a significant interaction between age and fluency modality, this result mainly reflects the relationship between action and phonemic fluency. We thus interpret that, in our cohort, semantic and phonemic fluency have a similar association with age. Other groups have also found that semantic and phonemic fluency have a similar association with age [1,39]. However, some studies have shown different results, as for example, an association between semantic fluency and age but not between phonemic fluency and age [13–17,48]. The age range investigated is a major confounder, accounting for part of these contradicting results. Importantly, by covering a wide range of age, from 32 to 84 years, our data help to further understand these discrepancies as well as to delineate the age dynamics in fluency performance.

### Differentiation, dedifferentiation, and stability patterns

The contribution of various cognitive functions to verbal fluency performance was different depending on the fluency modality and the age group. Regarding semantic fluency, in the middle-age group, the main contribution was seen for lexical access, processing speed, and verbal memory. In the early elderly group, we observed a greater contribution of executive functions, including working memory, in addition to verbal memory. Lexical access also contributed somehow but the contribution of processing speed was lesser than in the middle-age. Previous studies have found an association of semantic fluency with lexical access [24–26,28], processing speed [32], executive functions [28], and working memory [25]. The novelty of our study is that we reveal age-specific contributions of different cognitive functions to semantic fluency. It is very interesting that the contribution of executive functions was observed in the range of age with greater reduction in word production. This happened in a context of differentiation. This means that when several relevant cognitive functions stop contributing to semantic fluency (differentiation), performance in semantic fluency drops, but new executive components emerge (dedifferentiation), likely being recruited as a compensatory mechanism. This interpretation implies that both differentiation and dedifferentiation patterns can co-occur simultaneously from young ages and not only at the oldest ages, as previously suggested [37,38,51]. New brain networks or new parts of the same networks may be involved in this process, thus extending from the original differentiated function or network. According to the “Compensation-Related Utilization of Neural Circuits Hypothesis” (CRUNCH), the aged brain has to deal with processing inefficiencies and recruits more neuronal resources in order to achieve the same level of performance than a younger brain [52]. This hypothesis is further supported by our results obtained in the late elderly. Executive functions and memory functions remained to contribute to semantic fluency, and premotor functions emerged as a new contributor, also supporting the greater participation of the frontal lobe. The “scaffolding theory of aging and cognition” (STAC) [7] suggests a generalized increased frontal activation with age as a compensatory response. However, we found that the differentiation process is more prominent than the dedifferentiation process. This may be explained by the fact that executive functions can not completely compensate for the onslaught of aging; or this compensation coexists with the overall executive dysfunction observed in normal aging [53]; or both explanations at the same time.

Regarding phonemic fluency, lexical access, working memory, processing speed, and visuoconstructive abilities were the most important contributors in the middle-age. Verbal memory also contributed to a lesser extent. The contribution of visuoconstructive abilities may be explained by the strong executive component of the test used to measure this ability in our cohort. It is possible that shared processes such as planning and processing speed underlie both this test of visuoconstructive abilities [54] and phonetic fluency. The contribution of executive functions on phonemic fluency has been shown in previous studies [28,29,31]. In addition, premotor and visuospatial abilities emerged at the late elderly. Thus, new brain regions seem to be recruited as for semantic fluency (dedifferentiation), but possibly extending more to the posterior cortex in phonetic fluency. The visuoconstructive and visuospatial component of the tasks suggest a greater participation of the right hemisphere with increasing age, in line with the hemispheric asymmetry reduction postulated by the “hemispheric asymmetry reduction in older adults” (HAROLD) model [55]. The HAROLD effect observed by Cabeza (2002) [55] was interpreted as a compensatory function in which the brain additionally recruits homologous contralateral brain areas [56,57]. Our results suggest that this potential reorganization of the brain is rather effective, minimising the negative onslaught of aging on phonemic fluency, despite how challenging this task can be. This effectiveness contrasts with semantic and action fluency, where we observed a stronger association with age, perhaps due to less effective compensatory mechanisms and a more limited brain reorganisation. Potential explanations for this finding may be that phonemic fluency might be more relevant for the daily life, is more intensively trained during the lifespan, or category strategies are easier [24]. Alternatively, grammatical storages (semantic and actions) may be more vulnerable to aging, while phonemic fluency may allow more flexibility for the activation of different storages through switching strategies [13]. Other researchers have found an association of phonemic fluency with lexical access [29], memory function [31,32], and processing speed [25,26]. Again, the novelty of our study is that we reveal age-specific contributions of different cognitive functions to phonemic fluency.

The functions contributing the most to action fluency in the middle-age were executive functions, processing speed, and verbal memory. In addition to these, visual functions (visual memory and visuospatial functions) started contributing in the older age strata (dedifferentiation). This suggests that new brain networks or parts of the same networks are recruited, including more posterior and right hemispheric regions [55]. The same as for semantic fluency, this finding emerged in the age range with greater reduction in word production, possibly as a compensatory response. We are not aware of studies investigating the association of action fluency with cognitive functions other than episodic memory or picture naming [12,23].

Our interpretations in these last paragraphs regarding cognitive functions underlying different neuropsychological tests are based on the widely used classification of Lezak (2012) [21]. However, neuropsychological tests are known to tap on several cognitive functions, which may reflect that different cognitive functions partially share the same neuronal networks, a finding that would delineate the optimal organization of the human brain (the balance between differentiated and dedifferentiated cognitive components). This organization is adaptive to age-related brain changes and the share of neuronal networks will increase with aging as part of compensatory mechanisms (dedifferentiation).

The present study has some limitations. We analyzed cross-sectional data. Therefore, our age-related differences may partially be explained by cohort effects. We controlled for performance on WAIS-III Information as a means to control for generational effects often overlapped with crystallized intelligence [11,54]. Also, multivariate analysis methods such as random forest have been proven to maximize the covariance between the predictors and the outcome variable, being less vulnerable to confounders such as cohort effects [11]. Nonetheless, we are currently collecting follow-up data so that our present cross-sectional findings can be substantiated in a longitudinal design. In addition, future studies should further disentangle the mechanisms behind co-occurring differentiation and dedifferentiation processes. For example, how executive functioning substitutes the contribution of other cognitive functions, in a context of overall executive dysfunction as individuals age, needs to be further investigated. Fluency performance varies according to the type of stimulus (either letter or a category) [58]. Therefore, it is warranted to replicate our current findings using other stimulus for semantic (e.g. vegetables) and phonemic fluency (e.g. C-F-L). In this study we focused on high order cognitive functions. However, previous studies have shown that dedifferentiation findings extend to peripheral sensorimotor abilities such as visual and auditory acuity [59], which deserves further attention in the future. Also, we focused on the contribution of non-language functions (other than lexical access: BNT) towards the prediction of verbal fluency. Therefore, investigating the contribution of non-fluency language components towards the prediction of verbal fluency is warranted in future studies. The association between age and cognition is largely determined by biological changes taking place in the brain during aging. Therefore, extending our analyses to neuroimaging measures in the future is warranted and might help to better understand the neural correlates of our current findings.

### Conclusions

Verbal fluency declines with increasing age. Semantic fluency seems to be more vulnerable to aging than phonemic and action fluency. However, these dynamics are masked by the influence of other cognitive functions, which may themselves be declining with age as well. Lexical access, processing speed, and executive functions are among the most contributing functions. The most striking contribution of new cognitive functions takes place during the transition from the middle-age to the early elderly. Differentiation processes (functions stop contributing with increasing age) coexist with dedifferentiation processes (new functions start contributing with increasing age). Compensatory mechanisms are postulated to underlie these patterns. All in all, we present important data towards advancing to a better understanding of cognitive aging and compensatory processes. These findings may be relevant for personalizing age-specific cognitive interventions by guiding the development of materials for cognitive stimulation and/or rehabilitation in the close future. This knowledge may also be relevant for the clinical practice, improving interpretation of cognitive performance, and eventually improving diagnosis of cognitive disorders. Furthermore, our research could easily be extended to the study of other cognitive functions.

## METHODS

### Participants

A total of 446 participants were selected from the GENIC-database (Group of Neuropsychological Studies of the Canary Islands) [11], with ages between 32 and 84 years, and a balanced distribution of sex across age. All participants were evaluated with a comprehensive neuropsychological protocol, which assesses language, processing speed, attention, executive functions, verbal and visual episodic memory, procedural memory, and visuoconstructive, visuoperceptive and visuospatial functions (see Supplementary Table S1 and [60,61] for detailed information about the protocol). Inclusion criteria were: (1) normal cognitive performance in comprehensive neuropsychological assessment using pertinent clinical normative data (i.e. individuals with mild cognitive impairment or dementia were excluded); (2) preserved global cognitive and functional status operationalized as a Mini-Mental State Examination (MMSE) score ≥24, a Blessed Dementia Scale (BDRS) score <4 and/or a Functional Activity Questionnaire (FAQ) score <6); (3) no neurologic, psychiatric or systemic diseases; and (4) no history of substance abuse. An exception was done for BDRS. Although the BDRS scale cut-off for abnormality is frequently established at ≥4 points [62,63], the ‘changes in personality, interests and drive’ subscale may influence the BDRS total score and does not necessary reflect functional impairment. With the objective of excluding only individuals with functional impairment, we included those participants with total BDRS scores ≥4 (n=24) if: a) 70% or higher percentage of the BDRS total score resulted from the ‘changes in personality, interests and drive’ subscale; and b) if a score ≤1.5 was obtained in the other two subscales (‘changes in performance of everyday activities’ and ‘changes in habits’). The same procedure has been used in previous studies [11,64]. The study was approved by the ethics committee of the University of La Laguna (Spain) and all participants gave their written informed consent.

### Neuropsychological assessment

Among all the tests included in our neuropsychological protocol, three tests of verbal fluency are of special relevance for the current study:

### *Phonemic verbal fluency*

The Controlled Oral Word Association Test (COWAT [65];) was administrated. Participants had to recall words that begin with the letters F, A, and S, taking one minute on each of the letters. Proper nouns, numbers, and derived words were considered intrusion errors. A total score (F+A+S) was calculated as the number of correct words produced, excluding intrusions and perseverations (repetitions of correct words).

### *Semantic verbal fluency*

Instructions were given following the administration procedures described in the Multilingual Aphasia Examination [65]. Participants had to recall names of animals during one minute. The total number of words, perseverations, and intrusions were registered.

### *Action verbal fluency*

Participants had to recall verbs in infinitive form (e.g. “to reflect”). Verbs included as part of a sentence (e.g. “to dance the tango”) and repetitions of the same verb were considered errors [12]. The total number of correct verbs, intrusions, and perseverations were counted.

Other neuropsychological tests selected for this study are explained in Supplementary Table S1, including Information Subtest (from the WAIS-III), Boston Naming Test (BNT), PC-Vienna System, Paced Auditory Serial Addition Test (PASAT), Stroop Test, Trail Making Test (TMT), Colour Trial Test (CTT), Facial Recognition Test (FRT), Judgment of Line Orientation Test (JLOT), Digit Span (from the WMS-III), Visuospatial Span (from the WMS-III), Logical Memory (LM, from the WMS-III), “Test de Aprendizaje Verbal España-Complutense” (TAVEC, the Spanish adaptation and validation of the California Verbal Learning Test), Visual Reproduction (VR, from the WMS-III), Luria’s Premotor Functions (“hand alternative movements” and “motor coordination”), and Block Design (from the WAIS-III).

### Statistical analysis

Statistical analyses were performed using the R programming environment [66]. The association between age (between-subject factor, 3 or 4 age groups) and verbal fluency (within-subject factor, 3 fluency modalities) was tested using mixed ANCOVA, including WAIS-III Information as a covariable in order to control for between-subjects variability in the level of crystallized intelligence [11]. With the aim of investigating potential non-linear associations between age and performance in verbal fluency, we tested for quadratic and cubic associations in addition to linear associations in the mixed ANCOVA. To do this, we used the technique “trend analysis”, which is a way of decomposing the variance explained by the factor that accompanies an ANOVA using specially chosen linear weights called “orthogonal polynomials”. The polynomial contrast will test for trends in the data depending on the number of levels of the numeric factor. Since we have more than two levels in our independent variable, the polynomial contrast will examine other trends that can exist in the data such as quadratic and cubic trends. Random forest regression analyses were used to investigate the multivariate association between the measures of verbal fluency and a total of 45 cognitive variables. In random forest models, the contribution of the predictors in the models is reported as *Imp* (from *Importance*), which reflects the relative error in the prediction when a predictor is excluded from the model. *Imp* values higher than zero denote that a given variable contributes to the prediction of the outcome. The larger the *Imp* value, the greater the contribution. *Imp* values do not have an upper limit and they can rather be interpreted by considering the obtained values in relation to the variable yielding the highest *Imp* value in the model. Two per cent of the values were missing across the 48 cognitive variables and were thus imputed. Only the random forest analyses were performed on this imputed dataset. For the demographic variables, ANOVA was used for both continuous and dichotomous (dummy) variables. Simple regression analysis was performed to investigate the association between pairs of continuous variables. Significant differences were considered when p<0.05.

## Supplementary Material

Supplementary Tables
