# Supplementary material for: Cognitive compensatory mechanisms in normal aging: a study on verbal fluency and the contribution of other cognitive functions
Source: Aging (Albany NY). 2019 Jun 22;11(12):4090–106. doi: 10.18632/aging.102040 (PMC6628999; doi:10.18632/aging.102040)
Supplement: Supplementary Tables [file aging-11-102040-s001.pdf]

SUPPLEMENTARY TABLES

Table S1. List of neuropsychological tests grouped by cognitive functions.

Cognitive functions - Neuropsychological test (according to Lezak's (2012) classification)	Most prominent cognitive component
Global cognition and clinical variables	
Mini-Mental State Examination (MMSE) [1]	
Blessed Dementia Rating Scale (BDRS) [2]	
Functional Activity Questionnaire (FAQ) [3]	
Geriatric Depression Scale, Spanish version (GDS-VE) [4]	
Beck's Depression Inventory (BDI) [5]	
WAIS-III Information subtest (WAIS-III) [6]	
Processing Speed and Attention	
Choice Reaction Time – Motor and Reaction times (PC-Vienna System) [7]	cognitive and motor reaction times
Paced Auditory Serial Addition Test (PASAT) [8]	maintenance of attention
Trail Making Test-A (TMT-A) [9]	focusing/visual tracking
Color Trails Test - Part 1 (CTT-1) [10]	focusing/visual tracking
Visuospatial, visuoconstructive, and visuoperceptive functions	
Judgment of Line Orientation Test (JLOT, H form) [11]	visuospatial abilities
Facial Recognition Test (FRT-brief version) [11]	visuoperceptive abilities
Block Design – standard and extended version (WAIS-III) [6]	3-D visuoconstructive abilities
Visual Reproduction Test, Copy subtest (VRT, WMS- III) [12]	2-D visuoconstructive abilities
Visual Reproduction Test, Visual Discrimination subtest (VRT, WMS- III) [12]	visuoperceptive abilities
Working Memory, Executive Functions, and Premotor Functions	
Color Trail Test - Part 2 (CTT-2) [10]	mental flexibility/ executive control
Digit Span – forward and backwards (WMS-III) [12]	working memory: amplitude (forward) and manipulation (backward)
Visuospatial Span – forward and backwards (WMS-III) [12]	working memory: amplitude (forward) and manipulation (backward)
Stroop Test [13]	Sheet 1 Words and Sheet 2 Colors: processing speed<br>Sheet 3 Inhibition: executive function
Phonemic fluency – FAS (COWAT) [14]	
Semantic fluency – animals [14]	
Action fluency – verbs [15]	
Luria's Premotor Functions (Luria's) [16]	hand alternative movements and motor coordination
Learning and Memory	
Logical Memory (LM, WMS-III) [12]	Immediate recall, delayed recall, and recognition subtests (verbal)
Test de Aprendizaje Verbal España-Complutense (TAVEC, Spanish version of the California Verbal Learning Test (CVLT)) [17]	Immediate recall, delayed recall, and recognition subtests (verbal)
Visual Reproduction Test, (VRT, WMS-III) [12]	Immediate recall, delayed recall, and recognition subtests (visual)
Language	
Boston Naming Test (BNT) [18]	lexical access by visual confrontation

Table S2. Differentiation, dedifferentiation and stability patterns.

Pattern	Criterion
Differentiation pattern	A. The Importance of the predictor was higher than zero in the middle-age group but equal to zero in both the early elderly and the late elderly groups (i.e. gray-shaded cells in Table 3).<br>B. The Importance of the predictor was higher than zero in both the middle-age and early elderly groups but equal to zero in the late elderly group.
Dedifferentiation pattern	A. The predictor showed an Importance equal to zero in the middle-age group but did show an importance higher than zero in the early elderly and late elderly groups.<br>B. The predictor showed an importance equal to zero in both the middle-age and early elderly groups, but show an Importance higher than zero in the late elderly group. In
Stability pattern	A. The Importance of the predictors was higher than zero:<br>a. in the three age groups;<br>b. in the middle-age and late elderly groups;<br>c. only in the early elderly but with low Importance (an Importance value ≤25% of the highest Importance value within that model. For example, if the highest Importance values is 37, the cut-off value would be ≤9.25).<br>B. The predictors had an Importance value equal to zero in the three age groups.
Stable/differentiation pattern<br><br>and<br>Stable/dedifferentiation pattern	In some cases, we observed a combination of these patterns. For example, BNT shows both a stable/differentiation pattern, whereas CTT – Part 1 shows both stable/dedifferentiation pattern. Since this only affected a small percentage of the associations (all possible associations refers to number of variables (45) by three fluency modalities (45*3 = 135 associations)), for simplicity, we classified the stable/differentiation pattern as a differentiation pattern (7% of all associations), and the stable/dedifferentiation pattern as a dedifferentiation pattern (4% of all associations).<br>Based on the definitions described above, we calculated the percentage of variables that showed a differentiation, dedifferentiation, and stable pattern within each fluency modality (number of variables showing a given pattern / total number of variables).

Supplementary References

1. Folstein MF, Folstein SE, McHugh PR. "Mini-mental state". A practical method for grading the cognitive state of patients for the clinician. J Psychiatr Res. 1975; 12: 189–98.

2. Blessed G, Tomlinso BE, Roth M. Association between quantitative measures of dementia and of senile change in cerebral grey matter of elderly subjects. Br J Psychiatry. 1968; 114: 797–811.

3. Pfeffer RI, Kurosaki TT, Harrah CH, Chance JM, Filos S. Measurement of functional activities in older adults in the community. J Gerontol. 1982; 37: 323–9.

4. Martínez de la Iglesia J, Onís Vilches MC, Dueñas Herrero R, Albert Colomer C, Aguado Taberné C, Luque Luque R. Versión española del cuestionario de Yesavage abreviado (GDS) para el despistaje de depresión en mayores de 65 años: adaptación y validación. Medifam. 2002; 12: 620–30.

5. Beck AT, Ward CH, Mendelson M, Mock J, Erbaugh J. An inventory for measuring depression. Arch Gen Psychiatry. 1961; 4: 561–71.

6. Wechsler D. Wechsler Adult Intelligence Scale - Administration and Scoring Manual. 3rd ed. San Antonio, Texas: The Psychological Corporation; 1997.

7. Schuhfried G. Vienna Reaction Unit. Manual. Vienna: Schuhfried Ges.m.b.H.; 1992.

8. Gronwall DMA. Paced Auditory Serial-Addition Task: A Measure of Recovery from Concussion. Percept Mot Skills. 1977; 44: 367-73

9. Reitan RM. Validity of the trail making test as an indicator of organic brain damage. Percept Mot Skills.

1958; 8: 271–6.

10. D'Elia L, Saltz P. Color Trail 1 and 2. Odessa, FL: Psychological Assessment Resources; 1989.

11. Benton A, Hamsher S, Varney O, Spreen N. Contributions to neuropsychological assessment: A clinical manual. Press. OU, editor. New York; 1983.

12. Wechsler D. Wechsler Memory Scale – Third Edition Technical Manual. 3rd ed. San Antonio, Texas: The Psychological Corporation; 1997.

13. Golden CJ. Stroop Colour and Word Test: A manual for clinical and experimental uses. Chicago, Illinois: Stoelting Company; 1978.

14. Benton A, Hamsher K, Sivan A. Multilingual aphasia examination. Iowa City, IA: AJA Associates. 2nd Ed. Associates A, editor. Iowa City, IA: University of Iowa; 1989.

15. Piatt AL, Fields JA, Paolo AM, Koller WC, Tröster AI. Lexical, semantic, and action verbal fluency in Parkinson's disease with and without dementia. J Clin Exp Neuropsychol. 1999; 21: 435–43.

16. Christensen A. Luria's neuropsychological investigation. 2nd ed. Copenhagen: Munksgaard; 1979.

17. Benedet M, Alejandre M. TAVEC: Test de Aprendizaje Verbal España-Complutense. Manual. Madrid: TEA ediciones; 1998.

18. Kaplan EF, Goodglass H, Weintraub S. The Boston Naming Test. 2nd Ed. Philadelphia: Lea & Febiger; 1983.
